# Treatment of Severely Resorbed Maxilla Due to Peri-Implantitis by Guided Bone Regeneration Using a Customized Allogenic Bone Block: A Case Report

**DOI:** 10.3390/ma10101213

**Published:** 2017-10-21

**Authors:** Oliver Blume, Lisa Hoffmann, Phil Donkiewicz, Sabine Wenisch, Michael Back, Jörg Franke, Reinhard Schnettler, Mike Barbeck

**Affiliations:** 1 Private Practice, 80331 Munich, Germany; oliblume@aol.com (O.B.); mb@mkg-tal13.de (M.B.); 2 Botiss Biomaterials, 12109 Berlin, Germany; Lisa.Hoffmann@botiss.com (L.H.); Phil.Donkiewicz@botiss.com (P.D.); 3 Institut für Veterinär-Anatomie, -Histologie und -Embryologie, Klinik für Kleintiere, 35392 Giessen, Germany; Sabine.Wenisch@vetmed.uni-giessen.de; 4 Clinic for Trauma Surgery and Orthopedics, Elbe Kliniken Stade-Buxtehude, 21682 Stade, Germany; joerg.franke@elbekliniken.de; 5 Klinik und Poliklinik für Unfallchirurgie, Universitätsklinikum Gießen, 35392 Giessen, Germany; reiner.schnettler@mac.com; 6 Berlin-Brandenburg Center for Regenerative Therapies (BCRT), Charité-Universitätsmedizin, 13353 Berlin, Germany

**Keywords:** bone block, allograft, tissue reaction, Guided Bone Regeneration (GBR), CAD/CAM

## Abstract

The objective of this case report is to introduce a customized CAD/CAM freeze-dried bone allograft (FDBA) block for its use in Guided Bone Regeneration (GBR) procedures for severely deficient maxillary bones. Additionally, a special newly developed remote incision technique is presented to avoid wound dehiscence. The results show optimal integration behavior of the FDBA block after six months and the formation of new vital bone. Thus, the results of the present case report confirm the use of the customized CAD/CAM bone block for augmentation of complex defects in the maxillary aesthetic zone as a successful treatment concept.

## 1. Introduction

To date, the treatment of complex alveolar bone defects, especially within the aesthetic zone, remains challenging even with respect to both functional and aesthetic restoration. The clinician’s options for treating such defects used to be limited to the use of autologous bone grafts (ABG), with its known drawbacks of increased operation time, costs and complications; increased donor site morbidity and unpredictable resorption [1,2,3,4,5]. In recent years, the advancing development of bone substitute materials has created a set of alternatives with which comparably predictable clinical outcomes can be achieved [6], as they maintain comparable osteoconductive properties for ABG [7]. Freeze-dried bone allografts (FDBA) represent the most promising option because of low block graft failure rate, minimal resorption, and high implant survival rates [8,9,10].

Interestingly, the computer-aided design/computer-aided manufacturing (CAD/CAM) technology nowadays allows for patient-customized manufacturing of allogenic bone blocks for complex ridge augmentation. Since there is limited literature addressing the feasibility of this class of customized allogenic bone blocks, the objective of this case report is to introduce its use in Guided Bone Regeneration (GBR) procedures for severely deficient maxillary bones. Additionally, a special newly developed remote incision technique is presented to avoid wound dehiscence. Established histological and histomorphometrical analyses of the tissue reactions and the integration pattern of the FDBA material are included to show its remodelling capacities [11].

## 2. Case Report

**Figure 1:**
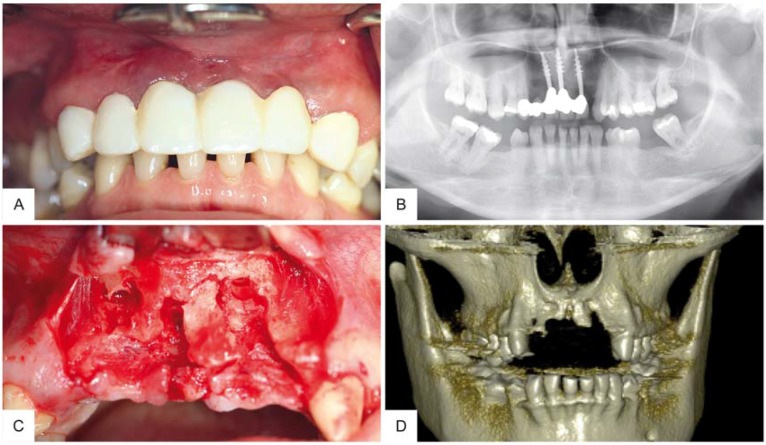
Clinical and radiographic examination of the maxillary defect. (**A**) Clinical preoperative examination revealed changed color in the gingiva on site #7–10; (**B**) Radiographic preoperative film demonstrated massive bone loss surrounding the three failing implants; (**C**) Complex bone defect and partial loss of the buccal wall within the maxillary aesthetic zone after extraction of failing implants. (**D**) CBCT image of the maxillary defect after implant extraction.

**Figure 2:**
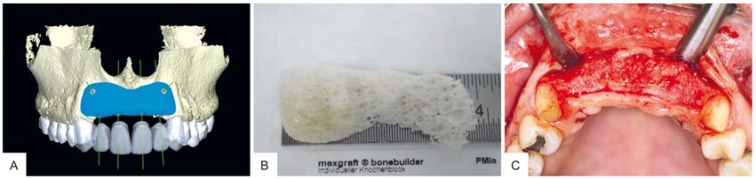
CAD/CAM block design and real bone allograft. (**A**) Virtual three-dimensional reconstruction of the defect and bone block design (blue); (**B**) Customized CAD/CAM bone block; (**C**) Grafted area showed sufficient bone volume and vital tissue for implant placement six months after GBR procedure.

A 43-year-old woman presented with the wish for a fixed prosthetic rehabilitation of the maxillary aesthetic zone. Preliminary clinical and radiographic evaluations showed peri-implantitis related to three dental implants with massive bone resorption and partial loss of the buccal wall within the maxillary aesthetic zone, tooth #7–#10 (ADA Dental Terminology 2011–2012) (Figure 1). The treatment plan agreed upon for this complex and spacious bone defect was to be a customized CAD/CAM freeze-dried bone allograft (maxgraft^®^ bonebuilder, botiss biomaterials GmbH, Zossen, Germany). After implant extraction and a healing period of three months, a cone beam computed tomography (CBCT) scan was taken and submitted in Digital Imaging and Communications in Medicine (DICOM) format to virtually design the allogenic bone block on a three-dimensional reconstruction of the patient’s defect (Figure 2). After review of the block design and approval by the surgeon, the customized FDBA block was milled from processed (Allotec^®^ process, Cells + Tissuebank Austria (C+TBA), Krems, Austria) cancellous bone of femoral heads of living donors.

## 3. Surgical Procedure

**Figure 3:**
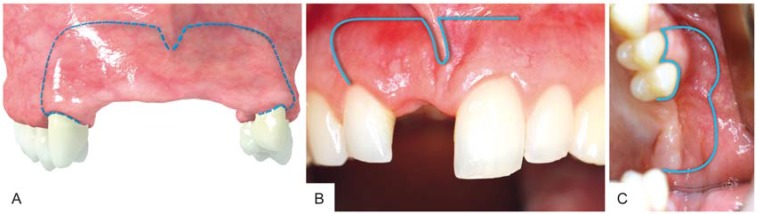
Remote incision techniques for augmentation procedures using a customized allogenic bone block. (**A**) Pillar incision performed as reported in this case. The horizontal part of the incision is positioned far up in the flexible mucosa in the vestibular fold and relief incisions are positioned in the posterior third of the adjacent teeth; (**B**) Semi pillar incision in case of a single tooth gap in the maxilla; (**C**) Lateral incision in case of a free end situation in the posterior maxilla.

Six months after extraction of the failing implants, a GBR procedure was performed under general anesthesia, including processing of autologous platelet-rich fibrin (PRF) matrices from patient’s blood and perioperative antibiotic prophylaxis (Clindamycin, 600 mg iv). After making a full-thickness remote ‘pillar incision’ (Figure 3), raising a vestibular flap with distal relief incisions on adjacent tooth #6 and #11, the buccal tissue was carefully dissected, protecting the neurovascular structures, and mobilized in palatinal direction for proper soft tissue management.

The cortical layer of the recipient site was perforated using a diamond bur to promote bleeding. Afterwards, the FDBA block was obtained sterile from the double blister package and rehydrated in exudate serum obtained during the PRF process by creating a vacuum in a disposable syringe. The block fitted exactly onto the recipient site, and was rigidly fixed on the maxillary ridge with 1.25 mm-diameter titanium osteosynthesis screws. Before fixation, a countersink for the screw heads was created using a diamond ball mill to avoid soft tissue perforation. The small residual volumes in mesial and distal areas were filled using allogenic cancellous bone substitute material (Human-Spongiosa CHB, botiss biomaterials GmbH, Zossen, Germany) and xenograft material (cerabone^®^, botiss biomaterials GmbH, Zossen, Germany), and sharp edges were smoothed. The surgical site area was covered with a resorbable barrier membrane of native pericardium (Jason^®^ membrane, botiss biomaterials GmbH, Zossen, Germany), which was fixed to the local bone using titanium pins, followed by one layer of PRF matrices. The grafted area was closed with a pulley suture for proper flap adaptation and to avoid any tissue strangulation by using absorbable 4.0/5.0 suture material. Sutures were removed in part 7 days, and entirely 14 days, postoperatively.

Six months after the GBR procedure, at re-entry, fixation screws were removed and bone core biopsies were taken for histological and histomorphometrical analysis based on previously described methods. In brief, the biopsies were fixed in 4% neutral buffered formalin for 24 h, decalcified in 10% Tris-buffered EDTA (Carl Roth, Karlsruhe, Germany) at 37 °C for 15 days and passed through a series of increasing alcohol concentrations followed by xylol. After embedding of the biopsies in paraffin, cutting was conducted using a microtome (Leica RM2245, Wetzlar, Germany) in sections of 3–5 μm thickness. Slides were stained with Masson-Goldner, Toluidin blue and a combinatory Safranin/Toluidin blue staining.

The histological examination included analysis of the following histological parameters: integration pattern of the graft, fibrosis, hemorrhage, necrosis, vascularization and the presence of neutrophils, lymphocytes, plasma cells, macrophages and multinucleated giant cells (MNGCs). The histological images were recorded by means of an Axiocam 105 color digital camera (Carl Zeiss AG, Oberkochen, Germany) connected to a computer system running the Zen software (version 2.3, blue edition, Carl Zeiss AG, Oberkochen, Germany). The histomorphometrical analysis included the following steps: initially, the histological slides were digitized using a light microscope (Axioscope 40, Carl Zeiss AG, Oberkochen, Germany) connected with a scanning table (EK 14 mot, Merzhauser, Wetzlar, Germany), a digital camera (AxioCam MRc 5, Carl Zeiss AG, Oberkochen, Germany) and a computer running the Zeiss AxioCam software (AxioVs40, version 4.8.2.0, Carl Zeiss AG, Oberkochen, Germany) at a 10× magnification. Total scans were used for histomorphometric measurements. Afterwards, the NIS Elements software (Basic Research, version 4.51, Nikon, Tokyo, Japan) was used for final image assessment. Finally, the graph was drawn using the software GraphPad Prism (Version 6.01, GraphPad Software Inc., La Jolla, San Diego, CA, USA).

**Figure 4:**
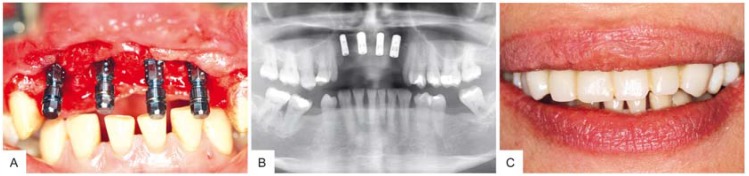
Four implants were placed in locations #7, #8, #9 and #10. (**A**) Buccal view after implant placement; (**B**) Radiograph taken immediate after the procedure; (**C**) Temporary restoration.

Four implants (Straumann Bone Level Roxolid^®^, Basel, Switzerland) were inserted under general anesthesia in locations #7, #8, #9 and #10 by the same surgeon who had performed the grafting procedure with a torque value of 25–50 N cm using a drill guide. Vestibuloplasty was performed using a 3D stable soft tissue collagenous graft (mucoderm^®^, botiss) and a radiograph was taken after implant insertion to confirm the correct implant position (Figure 4). Implants were uncovered three months after placement and again a radiograph was taken. The patient received temporary restoration and is awaiting final prosthetics sixteen months after grafting procedure.

## 4. Results

**Figure 5:**
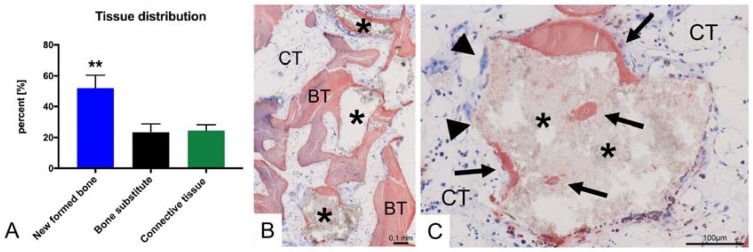
Results of the histological and histomorphometrical analyses. (**A**) Tissue distribution six months post-OP (** *p* > 0.01); (**B**) Integration of the FDBA material (asterisks) surrounded by vascularized connective tissue (CT) and new formed bone (BT); (**C**) Both the material-mediated bone growth (arrows) in combination with the multinucleated giant cells (arrowheads) resemble the ongoing remodeling processes.

The post-operative recovery and healing process was uneventful, and six months after GBR surgery, the grafted area showed sufficient bone volume and vital tissue for implant placement (Figure 2). Histologically vital new-formed bone was found in the augmentation area at re-entry, and the FDBA material was completely integrated within this new-built bone tissue, showing its optimal osteoconductive properties (Figure 5B). While much of the FDBA surface was covered by new-built bone tissue, a significant amount of the surface was covered by connective tissue containing multinucleated giant cells (Figure 5C). However, no histological signs of implant-related inflammation were observed (Figure 5C).

Histomorphometrical analysis showed that the amount of new bone (52%) was statistically higher (** *p* > 0.01) compared to the amounts of connective tissue (25%) and residual grafting material (23%), respectively (Figure 5A). Radiographs and clinical examinations at re-entry and during implant uncovering six and nine months after augmentation surgery indicated continuous remodeling of the allogenic bone block and hence stable osseointegrated implants providing an optimal result (Figure 2C and Figure 4).

## 5. Discussion

Allogenic bone blocks have several advantages over autologous bone blocks; namely, (1) no donor site morbidity, (2) no second surgical site, (3) less patient discomfort, and (4) reduced surgery time [12]. The added value of a precise fit gains importance for complex bone defects, as the space between residual bone and bone graft can be reduced to a minimum to enhance the physical contact between the graft and the recipient site to allow for FDBA revascularization through integration/replacement (creeping substitution) at the recipient site [13,14]. Moreover, this direct contact with the neighboring bone tissue allows for a fast bony integration.

Furthermore, the application of customized CAD/CAM allogenic blocks reduces the time in surgery to a new minimum level, as shaping of the block is no longer necessary, and chair time can be significantly reduced for both the patient and the surgeon. Moreover, a reduction in the time in surgery would be expected to reduce the infection rate of the recipient site and the graft, which is one major complication reported for the use of allografts [15,16]. In this context, the decrease of graft infection is expected based on the fact that CAD/CAM-designed bone blocks are no longer subjected to numerous possible sources of contamination during manual adjustment deriving from prolonged contact with the gloves of the surgeon, the oral fluids of the patient, the burs, and other environmental factors [16].

The present results show an optimal integration behavior of the FDBA block after six months and the formation of new vital bone, which is comparable to values when using other treatment options for such kind of defects [17,18]. Thus, the analyses of the present case report confirm the use of the customized CAD/CAM bone block for augmentation of a complex defect in the maxillary aesthetic zone as a successful treatment concept.

The rationale behind using the remote ‘pillar incision’ technique described herein is to produce a tension-free primary closure, because inability to obtain tension-free closure of the advanced flap can encourage incision line opening and membrane exposure, which are common complications following augmentations with cancellous block allografts [15]. Advantages of this remote technique include the following: (1) the incision is positioned far away from graft; (2) intact keratinized mucosa on the alveolar ridge and intact papillae; and (3) no visible scars, because the incision lies in the flexible mucosa in the vestibular fold. To deepen the understanding of this incision technique for GBR procedures using the customized bone block, two different cases (single tooth gap and free end situation in the maxilla) have been included to highlight the alternative incision line performed therein.

Although there are some reports that midcrestal incisions have the greatest anatomic potential for success in GBR procedures due to the features of mucosal vascularization of the alveolar ridge [19,20], the remote incision technique introduced in this case report has proven to be a valuable alternative, ensuring sufficient mobilization of the overlying soft tissue to cover the graft, resulting in uneventful wound healing with no aesthetic impairment in the maxillary aesthetic zone. However, further studies involving more cases are necessary to verify the reliability and validity of this technique.
